# Reversal of 5-fluorouracil resistance by EGCG is mediate by inactivation of TFAP2A/VEGF signaling pathway and down-regulation of MDR-1 and P-gp expression in gastric cancer

**DOI:** 10.18632/oncotarget.20666

**Published:** 2017-09-06

**Authors:** Hongsheng Tang, Lisi Zeng, Jiahong Wang, Xiangliang Zhang, Qiang Ruan, Jin Wang, Shuzhong Cui, Dinghua Yang

**Affiliations:** ^1^ Department of Hepatobiliary Surgery, Nanfang Hospital, Southern Medical University, Guangzhou 510515, Guangdong Province, China; ^2^ Department of Abdominal Surgery, Affiliated Cancer Hospital & Institute of Guangzhou Medical University, Guangzhou 510095, Guangdong Province, China

**Keywords:** EGCG, gastric cancer, drug resistance, 5-fluorouracil, VEGF

## Abstract

The effect of 5-fluorouracil (5-FU) chemotherapy for gastric cancer (GC) is limited by drug-resistance. To conquer this drug-resistance, various treatments including combination therapy have been used, but the overall survival has not been improved yet. In our current study, 5-FU resistant GC cells, SGC7901/FU and MGC803/FU, were established by long term exposure to 5-FU, and the proliferation capability of these resistant cells was verified to be reduced. The drug related proteins, MDR1 and P-gp were up-regulated in resistant cells compared to the parental cells. We further found proliferation and tumor growth suppressed effects of epigallocatechin gallate (EGCG), which is the predominant polyphenolic catechin constituent in green tea, on both the 5-FU resistant cells and the SGC7901/FU xenograft. Furthermore, an interesting results showed that reversal of 5-FU resistance of GC cells by EGCG treatment *in vivo* and *in vitro*. In the molecular study, We also found that EGCG suppressed the expression of both MDR-1 and P-gp at mRNA and protein levels *in vivo* and *in vitro*. Western blot and ELISA assay revealed that EGCG was able to inhibit VEGF secretion and expression, and its up-stream signal regulator, transcription factor activator protein 2A (TFAP2A) was also down-regulated by EGCG, our results indicated that TFAP2A/VEGF axis is one of the critical pathway inhibited by EGCG for cell proliferation and 5-FU resistance. Taken together, our data suggested that EGCG inhibits GC growth and reverses 5-FU resistance of GC through inactivation of TFAP2A/VEGF pathway and down-regulation of MDR-1 and P-gp expression.

## INTRODUCTION

Gastric cancer is one of the most common cancers worldwide and it is particularly prevalent in Asia [1]. Patients with early-stage gastric cancer have a good prognosis following medical or surgical treatment [2], but advanced or recurrent gastric cancer patients have high mortality rates, due to drug resistance [3]. Chemotherapy in gastric cancer is generally used in multimodality treatment, such as perioperative and adjuvant chemotherapy. The common chemotherapeutical agents for gastric cancer include cisplatin (CDDP), 5-fluorouracil (5-FU) or its oral administered derivatives capecitabine. In the past decades, the therapeutic regimen of docetaxel + cisplatin + 5-fluorouracil has been used as a first line treatment for advanced gastric cancer [4, 5]. However, for patients with advanced gastric cancer, the response rate to these chemotherapy is more than 50% and nearly all patients develop chemotherapy resistance [6, 7]. Obviously survival benefits of 5-FU based chemotherapy have been reported in patients with metastatic gastric cancer [8]. Although such regimens have improved response, many patients have recurrence after several courses of 5-FU based chemotherapy [9]. The drug-resistantance of certain tumors to 5-FU therapy is thus a major clinical problem, but the molecular mechanisms underlying the development of 5-FU chemo-resistance in patients with cancer remains poorly understood.

To avoid 5-FU chemo-resistance for gastric cancer treatment, combination therapy has been used, but the total survival rate is not significantly improved [10]. The drug-resistant related proteins (DRPs), such as GST-π, MDR-1, P-glycoprotein (P-gp), and ABCG2 [11, 12], which mediate related signaling pathways that have been reported to inhibit apoptosis, promote proliferation, and induce drug resistance [13]. Of note, high expression of MDR-1, P-gp, ABCG2 and GST-πwas found in many tumor cells and tissues including gastric, lung, colorectal, breast and prostate cancers [13–17]. Hence, DRPs proteins are deeply involved in tumorigenesis, development, progression, matastasis and drug resistance. DRPs protein could be a therapeutic target and a potential biomarker for diagnosis of cancer.

Epigallocatechin-3 gallate (EGCG), a potent anti-inflammatory molecule found in green tea. EGCG has been known as an effective anti-inflammatory, anti-oxidant, and anti-cancer drug [18–20]. EGCG has been recognized as an important chemopreventive agent and as a modulator of tumor cell response to chemotherapy to suppress the growth, invasion, metastasis and angiogenesis of various tumors by arresting the cell cycle, inducing apoptosis, targeting angiogenesis [21, 22], metastasis [23] and many other cellular regulatory pathways [24]. Many studies have reported many molecular mechanisms that explain its biological effect, apoptosis induction and regulation of angiogenesis, such as the VEGF receptor 2 and VEGF, and binding with Bcl-xLor Bcl-2 [24–27]. Recently, EGCG has also been shown to inhibit cell proliferation and drug-resistance in breast cancer through suppression of STAT and P-glycoprotein signaling pathways [28, 29]. However, unlike several other plant based drug therapies, whether EGCG can inhibit tumor growth of GC and contribute to sensitization against chemotherapeutic agent 5-FU remains unexplored.

Herein, in the current study, 5-FU resistant GC cells, SGC7901/FU and MGC803/FU, were developed by long term exposure to 5-FU. The proliferation potetial of these 5-FU resistant cells was verified to be receded. The drug related proteins, MDR1 and P-gp were up-regulated in the resistant cells lines contrast with its parent cells. We also showed that EGCG inhibited cell proliferation and reversing the 5-FU resistance of gastric cancer through inactivation of TFAP2A/VEGF signaling pathway.

## RESULTS

### Establishment of the 5-fluorouracil resistant gastric cancer cells

**Figure 1:**
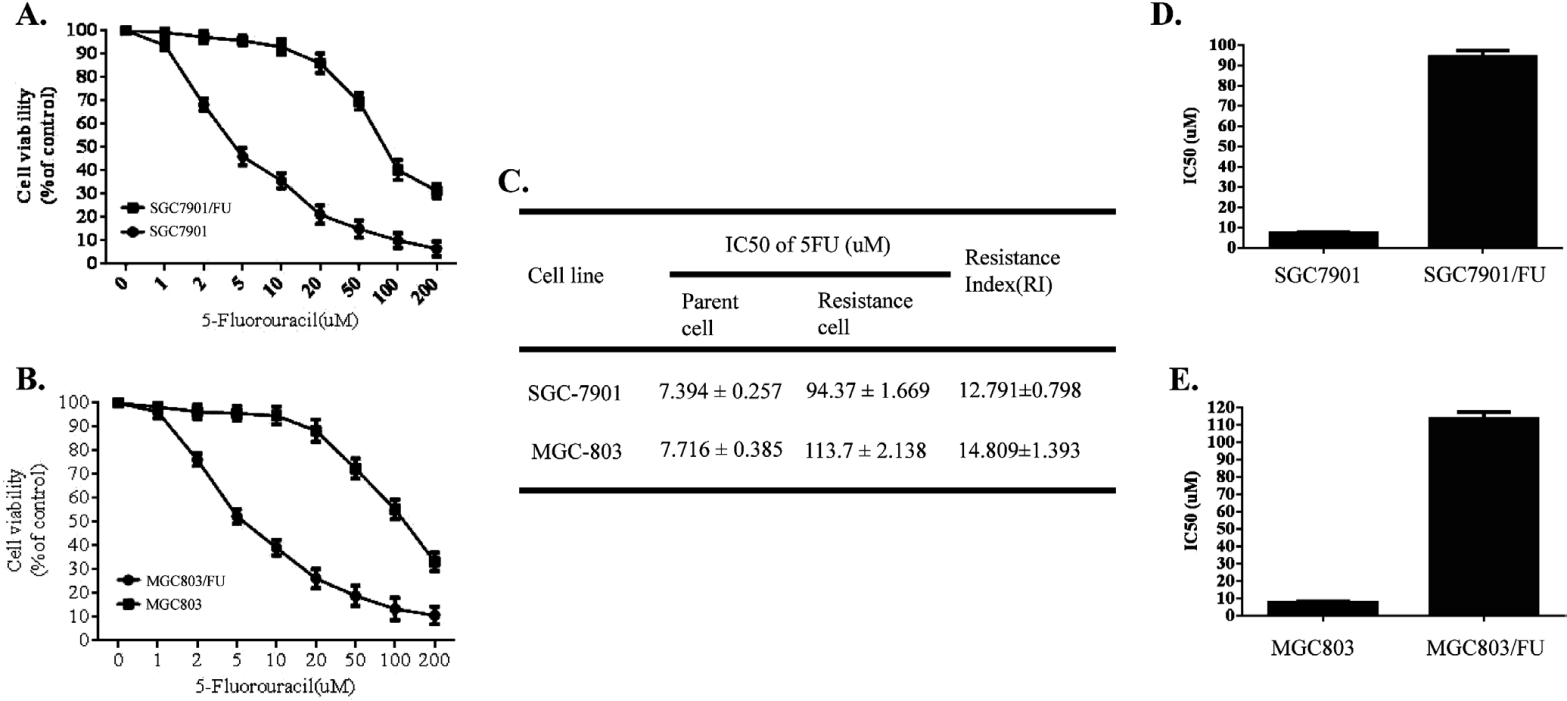
Sensitivity of the parental gastric cancer cells (SGC-7901 and MGC-803) and their 5-fluorouracil (5FU) resistant cell lines All cell lines were cultured with various concentrations (1,2,5,10,20,50,100 and 200uM) of 5-FU for 72 h. Cell proliferation activity was detected by CCK8 assay. Each data point represents the mean ± SD (n = 3). All 5-FU resistant cell lines were more resistant to 5-FU than the parental cell lines. **(A)** Sensitivity of the parental gastric cancer cell (SGC-7901) and its 5-fluorouracil resistant cell lines (SGC-7901/FU). **(B)** Cell proliferation activity of the parental gastric cancer cell (MGC-803) and its 5-fluorouracil resistant cell lines (MGC-803/FU). **(C)** Resistance Index of the parental gastric cancer lines and their 5-fluorouracil resistant cell lines to 5-FU. **(D)** IC50 value of the the parental gastric cancer (SGC-7901) and 5-fluorouracil resistant gastric cancer cell lines SGC-7901/FU. **(E)** IC50 value of the the parental gastric cancer (MGC-803) and 5-fluorouracil resistant gastric cancer cell lines MGC-803/FU.

To explore the molecular mechanism of 5-Fluorouracil(5-FU) resistance for gastric cancer, we developed the 5-Fluorouracil resistant gastric tumor cells, SGC-7901/FU and MGC-803/FU, by exposing the parental SGC-7901 and MGC-803 cells to 5-FU in a gradually increasing concentrations for 6 months. The proliferation potential and its morphological characteristics of these drug-resistant cells and their parental cells was analysised by cell activity and optical microscope. Results shown in Figure 1, 5-Fluorouracil resistant cells and parental cells were treated with 1,2,5,10, 20, 50,100,or 200 μM 5-Fluorouracil for 72 hours. The cell viability was detected by CCK-8 analysis. Results shown in Figure 1A and 1B, the cell viability of both resistant and parental cell was down-regulated in dose dependent manner. But, upon higher than 10 μM to 20 μM 5-Fluorouracil, the parental gastric cancer cells, SGC-7901 and MGC-803, were more sensitive to the 5-Fluorouracil than their resistant cells, SGC-7901/FU and MGC-803/FU.

The half inhibitory concentration (IC50) and Resistance Index(RI) of each cell lines for 5-FU were calculated. Interestingly, we found that the resistant cell lines SGC-7901/FU and MGC-803/FU have a higher IC50 value than their parental cells (Figure 1D and 1E). In addition, Resistance Index(RI), which reflected potential of drug resistance of each cell lines for 5-FU rose from 13 to 15 folds(Figure 1C). Our date indicated that SGC-7901/FU and MGC-803/FU cell lines acquired chemo-resistance potential.

**Figure 2:**
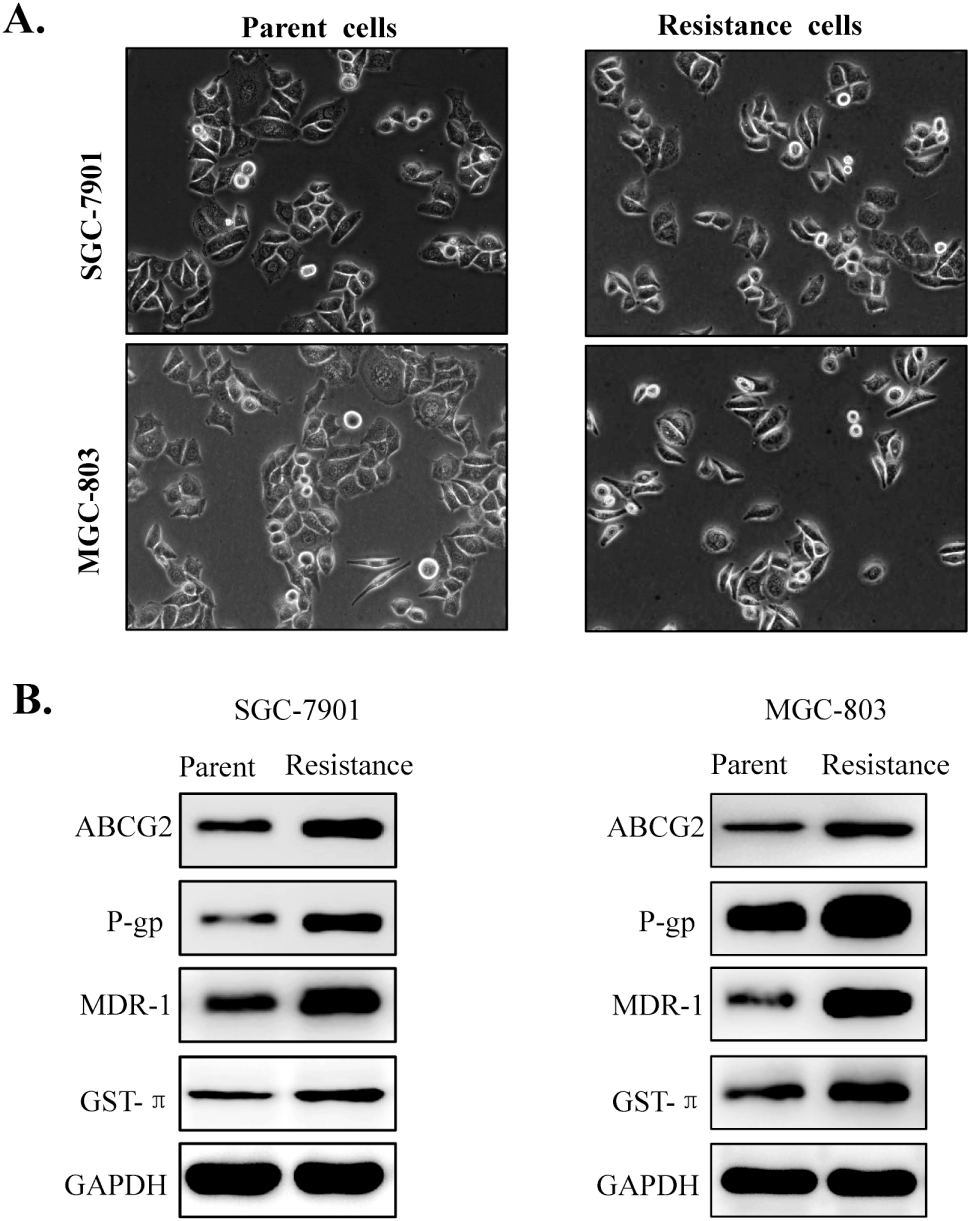
Morphological changes and drug resistance related proteins expression in the parental gastric cancer cells and its 5-fluorouracil resistant cell lines **(A)** Morphological changes of the drug resistant gastric cancer cells (SGC-7901 and MGC-803) compared with their parent cell lines (SGC-7901/FU and MGC-803/FU). **(B)** Drug resistance related proteins (ABCG2, MDR-1, GST-π and P-gp) were up-regulated in the the 5-fluorouracil resistant gastric cancer cell contrast to the parental cell lines.

Furthermore, we also evaluated morphological changes and drug resistance related proteins expression by western blot in the parental gastric cancer cells and their resistant cell lines. Result showed that there were no apparent morphological changes in the resistant cells compared with the parental cells (Figure 2A). In addition, the expression of ABCG2, MDR-1,p-GP and GST-π, four key drug resistance related proteins involved in 5-fluorouracil (5-FU) resistance for multiple cancer cells, were significantly increased in the resistant cell lines(Figure 2B). All the data confirmed that 5FU resistant gastric cell lines were established successfully.

### EGCG inhibited proliferation of 5-fluorouracil resistant gastric tumor cells

**Figure 3:**
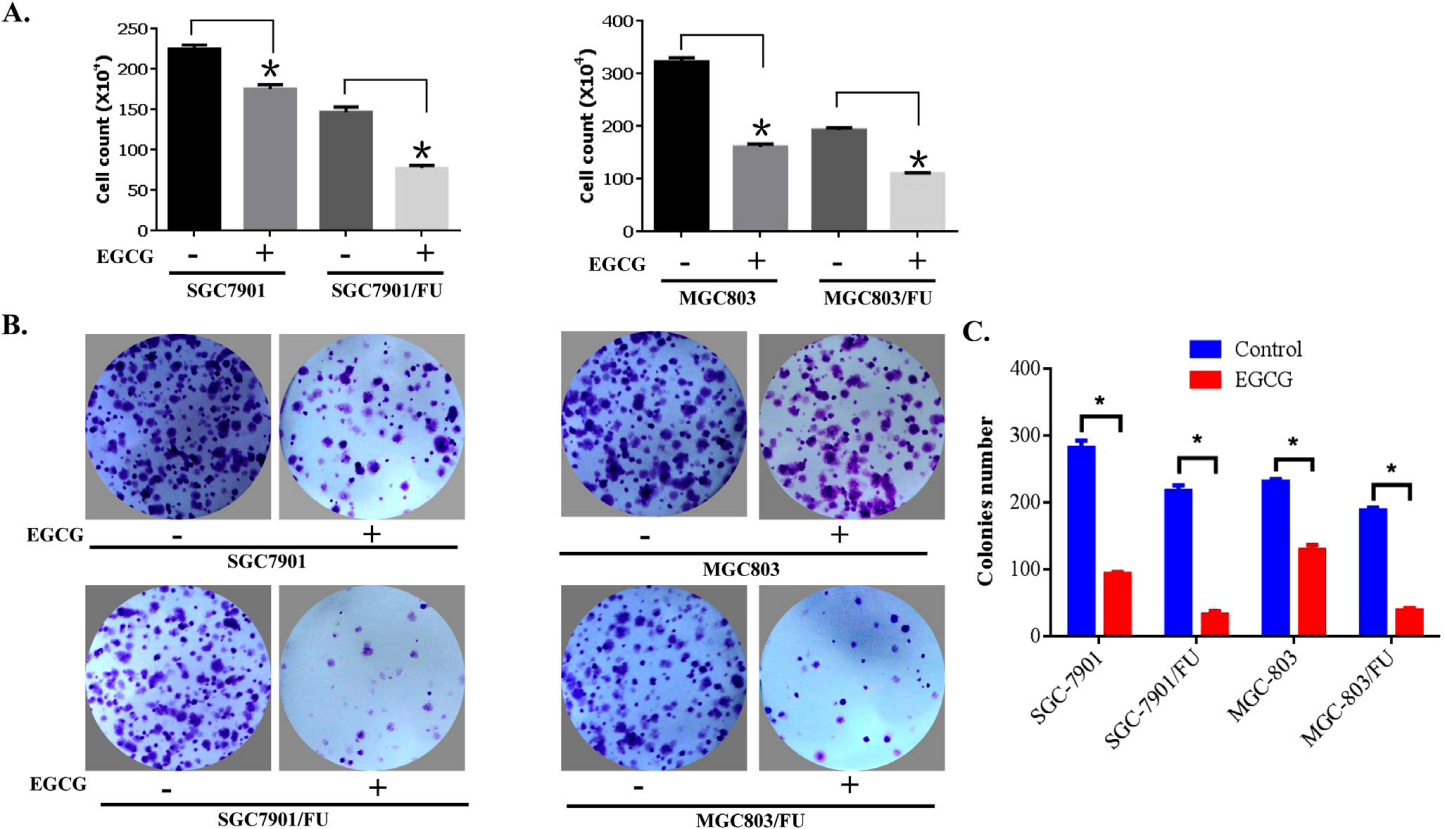
EGCG inhibited proliferation of both parental and 5-FU resistant gastric cancer cells **(A)** Gastric cancer cells (3×10^3^cells/well) were seeded onto 96-well plates, grown overnight and treated with 20 μM EGCG for 72 hours. After treatment, cells were harvested, stained with trypan blue, and the number of viable cells was counted. Data are presented as mean±SD of at least three independent experiments. The asterisks indicate the significant difference compared to the control value. **(B)** Gastric cancer cells were seeded onto 6-well plates (400 cells/well) and allowed to grow in the absence or presence of 10μM EGCG for 10 to 14 days. The colonies were visualized by crystal violet staining. **(C)** Quantitative analysis of the colony number in the different treatment groups. The data are presented as mean ± S.D. of three separate experiments. * P<0.05, the significant differences between treatment and control groups.

EGCG has been found to have anti-proliferation effect on many tumor cells [24], we explored if it could inhibits the proliferation of 5-Fluorouracil resistant cells. gastric cancer cells (3×103cells/well) were seeded onto 96-well plates, and treated with 20μM EGCG for 72h. The treatment cells were harvested, stained with tryphan blue, and we then counted the number of viable cells. We discovered that EGCG inhibited the cell viability of both 5-Fluorouracil resistant and parental cells (Figure 3A). The anti-proliferation action of EGCG seems to be more prominent in 5-Fluorouracil resistant cells than their parental. Furthermore, the anti-proliferation effect of EGCG on gastric cancer cells was carryed out by Colony-forming assay. As shown in Figure 3B upper panel, the parental cells, SGC-7901 and MGC-803, disply a obviously colon formation inhibition. But for while SGC-7901/FU and MGC-803/FU cells almost failed to form colony in the EGCG treatment group (Figure 3B lower panel). our results indicated that EGCG inhibited colony formation and cell proliferation of both 5-Fluorouracil resistant and parental cells. More importantly, the anti-proliferation potential of EGCG for the resistance cells was stronger than the parent cells.

### TFAP2A/VEGF signaling pathway was involved in the anti-proliferation effect of EGCG

**Figure 4:**
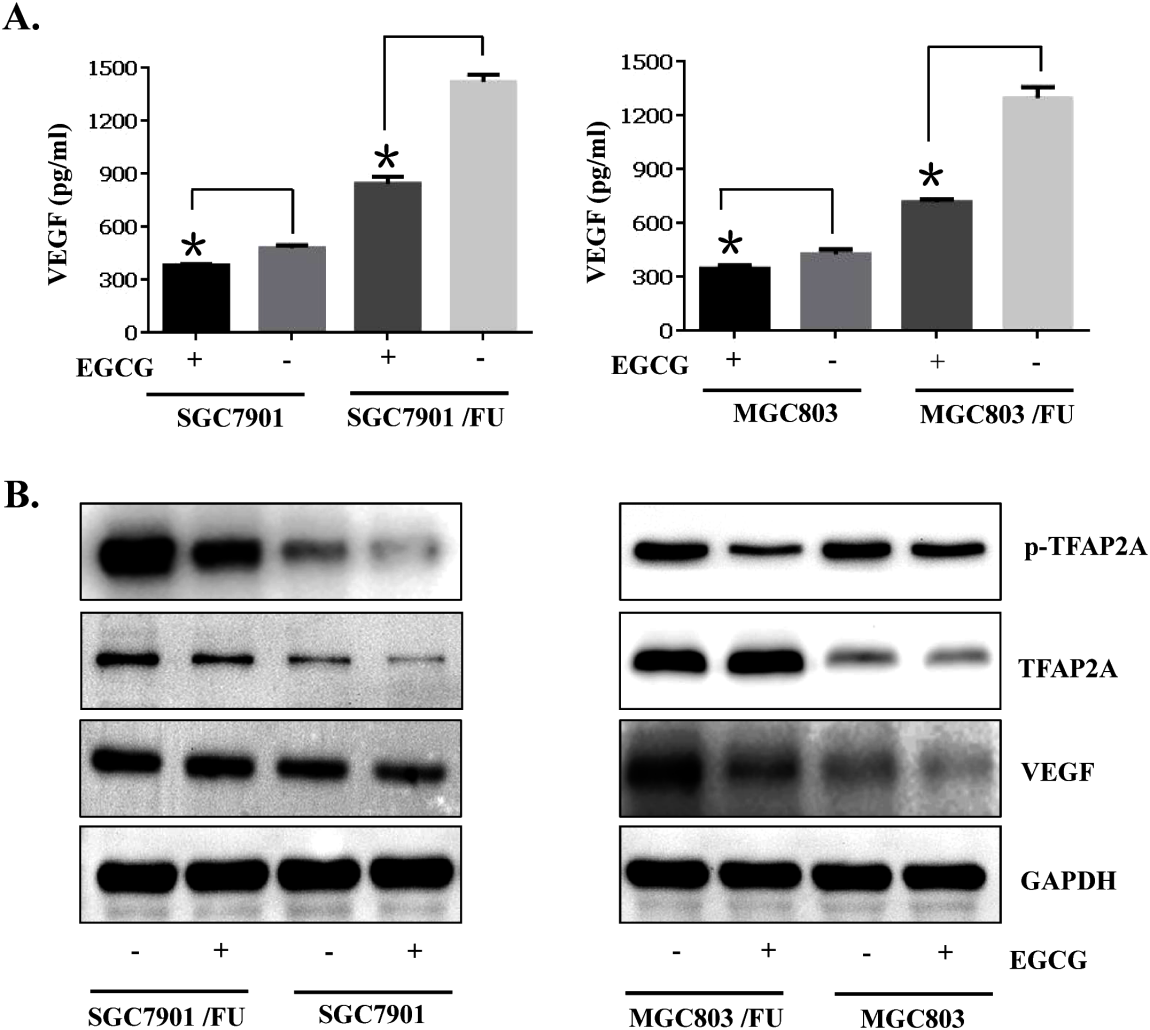
TFAP2A/VEGF signaling pathway was inactivated by the anti-proliferation effect of EGCG in the parental and 5-FU resistant gastric cancer cells Gastric cancer cells (SGC7901 and MGC803) and their resistant cells (MGC803/FU and SGC7901/FU) were seeded onto 100 mm dishes (2×10^5^cells/dish), grown overnight and treated with 20μM EGCG for 48 hours. **(A)** Conditioned media were harvested and used for VEGF assay by ELISA. To assess its production per well, the total amount of VEGF was normalized by the number of viable cells. Data are presented as the mean±SD of triplicate samples from three independent experiments. **(B)** Western blot analysis was performed to determine the phosphorylation status of TFAP2A, the level of total TFAP2A, the TFAP2A target genes VEGF, and of GAPDH as a loading control, was also detected. The data shown are representative of three independent experiments. The data in panel (A) are presented as the mean ± SD. The level of significance was indicated by P<0.05.

Transcription factor activator protein 2 family proteins play a pivotal role in cancer cell proliferation, cell migraton, cell invasion, tumor metastasis and chemo-resistance [30–34]. In many tumor cells, High expression of vascular endothelial growth factor (VEGF) expression and secretion is observed. VEGF expression known to be resulted from the up-regulation of upstream regulator TFAP2A [30]. We detected the inhibition of gastric cancer cell proliferation by EGCG, and which related to its regulatory action on VEGF expression, secretion and TFAP2A expression. Enzyme-linked immunosorbent assay result showed that VEGF secretion in the cell culture media was a bit down-regulated when EGCG treatment in the resistant SGC-7901/FU cells (Figure 4A). our data also showed that EGCG treatment had no signally action on the total TFAP2A expression in both 5-Fluorouracil resistant cells and parental by immunoblot assay(Figure 4B). However, consistent with the decreased VEGF secretion from gastric cell, phosphorylation of TFAP2A was also significantly decreased in 5-Fluorouracil resistant cells for treatment with EGCG in the parental and resistant gastric cancer cells(Figure 4B). Taken together, our results indicated that TFAP2A/VEGF signaling pathway was involved in EGCG-induced growth inhibition of 5-Fluorouracil resistant gastric cells.

### EGCG inhibited P-gp and MDR-1 proteins expression

**Figure 5:**
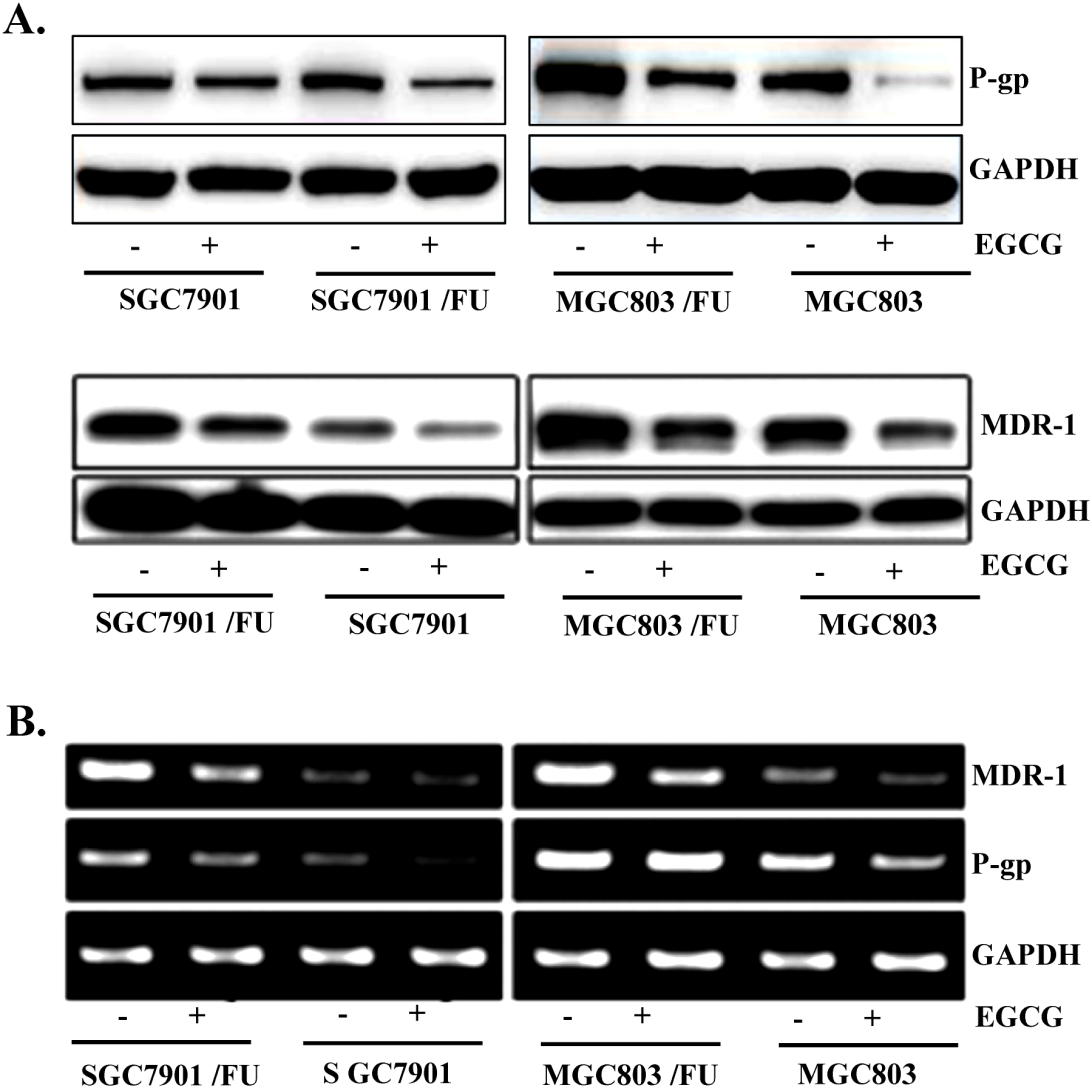
EGCG suppressed expression of MDR-1 and P-gp in both the parental and 5-FU resistant gastric cancer cells Gastric cancer cells (SGC7901 and MGC803) and their resistance cells (MGC803/FU and SGC7901/FU)were seeded onto 100 mm dishes (2×10^5^cells/dish), grown overnight and treated with 20 μM EGCG for 48 hours. **(A)** The protein level of MDR-1 and P-gp was assessed by Western blot analysis to determine the effect of EGCG on their expression. GAPDH was used as a loading control. Results are from three independent experiments. **(B)** For RT-PCR assay, total RNAs from those cells were isolated and used for analysis of MDR-1 and P-gp mRNA expression. The level of MDR-1 and P-gp mRNA was normalized to that of GAPDH. The data shown are representative of three independent experiments.

Since drug-resistance related proteins, P-gp, MDR-1 and ABCG2 have been known to promote tumor cell growth and drug resistance [10]. we detected if EGCG affected the expression of drug-resistance related proteins. Among DRPs members, both MDR-1 and P-gp expressions were examined at protein and mRNA level after exposure of cells with 30 μM EGCG for 48 h. Western blot analysis showed that EGCG treatment caused significant reduction of both MDR-1 and P-gp protein level in both parental and 5-Fluorouracil-resistant cells(Figure 5A). In addition, down-regulation of MDR-1and P-gp mRNA expression in EGCG-treated cells was confirmed by RT-PCR. As shown in Figure 5B, the mRNA levels of MDR-1 and P-gp were decreased by EGCG treatment, which was consistent with Western blot results. Of note, both MDR-1 and P-gp expression in 5-Fluorouracil resistant cells was dramatically reduced, compared with that in their parental cells, indicating that EGCG inhibited expression of MDR-1 and P-gp drug-resistance related proteins.

### EGCG could synergize with 5-FU to inhibit the growth of gastric cancer xenograft

**Figure 6:**
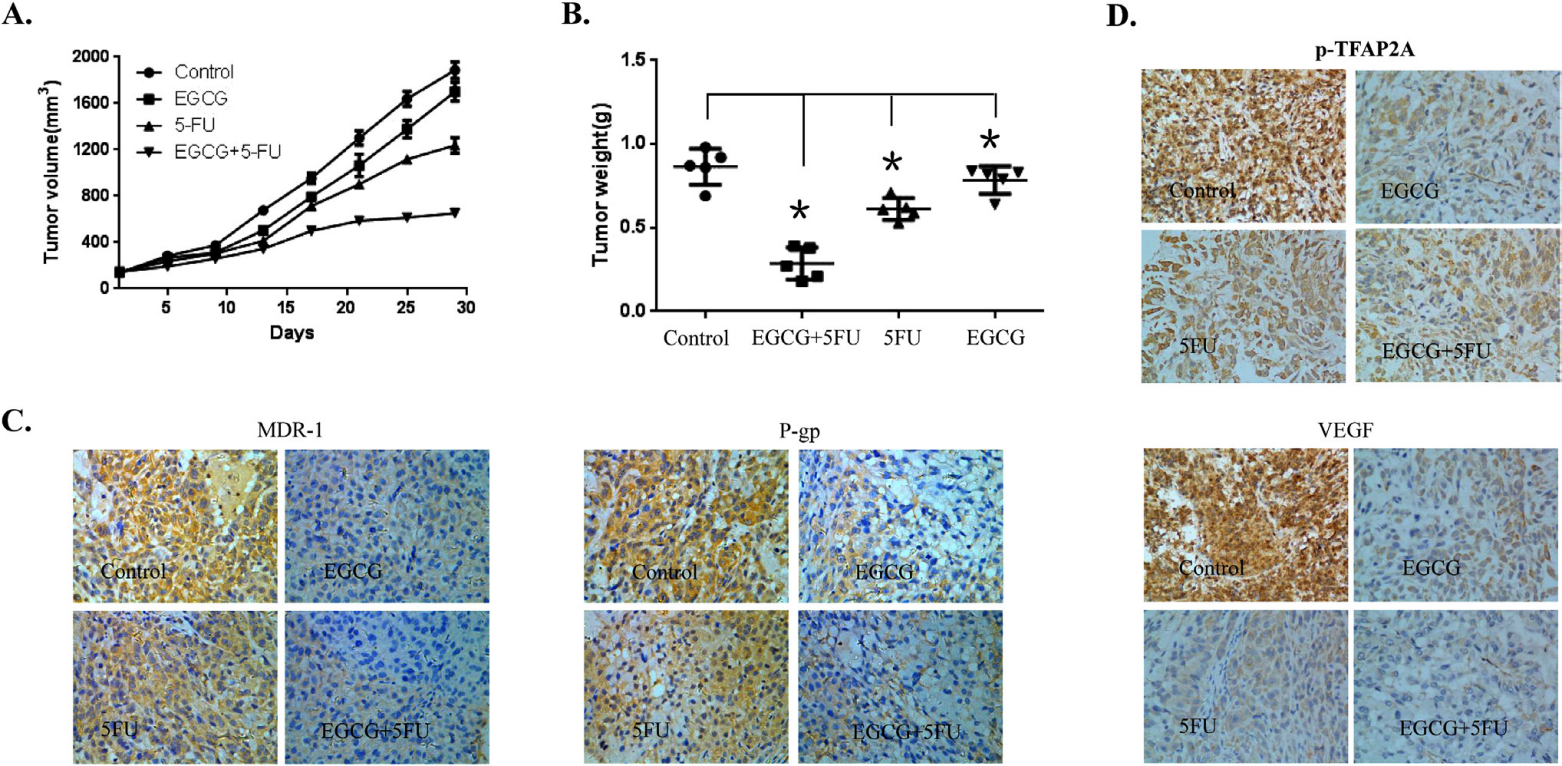
Effect of EGCG and 5-FU combination on tumor growth in a xenograft mouse model of human gastric cancer The female athymic nude mice aged 4 to 6 weeks were used in the study. SCG7901/FU cells (5 × 10^6^ in 100 μl PBS) were injected subcutaneously into the left flank of each mouse. When the formed tumor reached 150 mm^3^ after cell inoculation, the animals were divided randomly into four groups with 5 mice in each group. One group were intratumorally injected with PBS as the control, the second group received EGCG (25 mg/kg) treatment alone, the third group received 5-FU (20 mg/kg) treatment, and the fourth group received 5-FU and EGCG co-treatment. The mouse body weight and tumor volume were measured twice a week. The tumor volume was calculated as follows: V=(width^2^×length)/2. At the termination of the experiment, the mice were sacrificed and the tumors from each mouse were excised, and the tumor volume **(A)** and tumor weight **(B)** were calculated. The expression of MDR-1, P-gp, p-TFAP2A and VEGF proteins in tumor tissues was analyzed by IHC staining **(C** and **D)**. The data in panels (A-B) are presented as the mean ± SD. The level of significance was indicated by P<0.05. N=5 mice/group. Magnification, 200X.

Based on the results from *in vitro* studies, we further investigated the potential of the combined treatment with EGCG and 5-FU as a novel molecular therapeutic agent for tumor growth in a human gastric cancer xenograft mouse model. The SGC7901/FU cells were injected subcutaneously into the left flank of nude mice, and visible tumors developed at the injection sites after eight days with a mean tumor volume of 150 mm3. Mice were randomly divided into four treatment groups. After administration with EGCG or 5-FU alone or the two together for 30 days, and the tumors of each treatment group were peel off, and the tumor volume (Figure 6A) and tumor weight (Figure 6B) were monitored respectively. Results shown that the tumor volume and tumor weight were significantly inhibited by EGCG or 5-FU alone. However, co-treatment with EGCG and 5-FU together dramatically inhibited the growth of xenograft as compared with the treatment with 5-FU or EGCG alone(Figure 6A and 6B). In addition, the combined treatment did not significantly affect body weight of the mice (date not shown).

Furthermore, the immunohistochemical staining analysis of tissue of the xenograft also showed that co-treatment with EGCG and 5-FU in the mice enhanced the suppression of some key proteins involved in drug resistance, including MDR-1, and p-GP proteins (Figure 6C). Moreover, the immunohistochemical staining assay was also used to determine the expression of VEGF and p-TFAP2A. The expression levels of VEGF and p-TFAP2A were significantly decreased, by the combined treatment with EGCG and 5-FU as compared with the control group (Figure 6D). These results supported that EGCG could synergize the effect of 5-FU to inhibit the growth of human gastric cancer xenograft by inactivation of the TFAP2A / VEGF signaling pathway and down-regulation of drug resistance related proteins.

## DISCUSSION

5-Fluorouracil chemotherapy is the first line therapeutic drug for gastric cancer. Yet, the drug-resistance to 5-Fluorouracil restricts its anti-tumor action in clinical. To understand the drug resistance molecular mechanisms of 5-Fluorouracil involved in gastric cancer, we successfully established the 5-Fluorouracil resistant gastric cancer cell lines, SGC-7901/FU and MGC-803/FU. Our data indicated that the proliferation rate of these resistant cells was found to be much lower than their parental cells, and also, expression of drug resistance related proteins GST-π, MDR-1, P-gp and ABCG2 were signifcantly up-regulation in the resistant cells than their parents. Our current results indicated that long term exposure 5-Fluorouracil with a gradually increasing concentrations seems to be a strategy to establish the 5-Fluorouracil resistant gastric cancer cells.

The synergistic anti-cancer effect of EGCG in combination with other anti-tumor agents such as CDDP, docetaxel, 5-fluorouraciland paclitaxel on various tumors in many reports [35–38]. Especially, recent research showed that co-treatment of CDDP and EGCG induced apoptosis of resistant ovary and lung cancer cells by trargeting expression of the CTR1 [39, 40], these report suggest that EGCG could be a useful drug to conquer chemo-resistance in cancer cells. Consistent with these reports, our study found that EGCG was able to restrain gastric cancer cell proliferation with its 5-Fluorouracil resistant cells and tumor growth *in vivo*.

Transcription factor activitor protein 2 family proteins play a important role in tumor growth, cell proliferation, cell migration, cell invasion, tumor metastasis and chemo-resistance [30, 32–34]. In a wide variety of cancer, high expression of vascular endothelial growth factor (VEGF) and secretion was reported.

which known to be resulted from the increased of its upstream regulators hif-1αand the Transcription factor activitor protein 2A(TFAP2A) protein [30]. In our study, We found that EGCG inhibited VEGF expression and secretion, but did not significantly affect total TFAP2A expression levels. However, our results shown that phosphorylation of TFAP2A was obviously surppressed in 5-Fluorouracil resistant cells by EGCG treatment, suggested that activation of TFAP2A/VEGF pathway seems to be one of the molecular mechanisms which was produced during gastric cancer cells resistance acquisition.

Among DRP protein members, ABCG2, P-gp and MDR-1 over-expression has been reported in many cancers and drug resistance cells. Of note, the expression of ABCG2, P-gp and MDR-1 was found to be increased in gastric cancer cell lines [6, 8, 12, 13]. The clinical importance of MDR-1 was also detected by the report that in most of gastric cancer tissues. Protein level of MDR-1 was high and match with distant metastasis as well as lymph node metastasis [41]. Targeting of MDR-1 with specific RNA interference or monoclonal antibodies has been shown to restrain cancer cell proliferation and tumor growth [42]. In our study, We found that MDR-1 expression was suppressed in 5-Fluorouracil resistant cells by EGCG, elaborate on its reduced of cell proliferative potentail. Furthermore, we also discovered, EGCG treatment inhibited expression of MDR-1 at transcriptional level. Our results indicated that such inhibitory action of EGCG on MDR-1 expression seems to be associated with its transcription regulate function. It should be further research the molecular mechanisms in the future work.

In a words, 5-FU resistant GC cell lines, SGC7901/FU and MGC803/FU, were established by exposure to 5-FU for a long term, and the proliferative potentail of the 5-FU resistant cells was proved to be decreased, The drug related proteins, such as, MDR1 and P-gp were up-regulated in the resistant GC cells lines contrast with its parental cells. We found proliferation inhibition and tumor growth supression effects of EGCG, on both the 5-FU resistant cells and the xenograft mouse model. Furthermore, an interesting result shown that reversal of 5-FU Resistance to GC cells by EGCG treatment *in vivo* and *in vitro*. In the molecular mechanism study, we also found that EGCG suppressed the expression of both MDR-1and P-gp drug-resistance related proteins at mRNA and protein level *in vivo* and *in vitro*, Western blot and ELISA assay revealed that EGCG was able to inhibit VEGF secretion and the expression of transcription factor activator protein 2A phosphorylation, indicating that TFAP2A/VEGF axis was inhibited by EGCG for gastric cancer cell proliferation and 5-FU resistance. All in a word, our datas have revealed that EGCG inhibits GC growth and reversal of fluorouracil resistance of gastric cancer cells through inactivation of TFAP2A/VEGF signaling pathway and down-regulation of MDR-1 and P-gp expression.

## MATERIALS AND METHODS

### Cell culture

SGC-7901 and MGC-803 cells were purchased from the American Type Culture Collection. Both cells were grown in RPMI 1640 (HyClone, Logan, UT) containing 10% FBS(HyClone, Logan, UT), 2 mM L-glutamine, 10 U/ml penicillin, and 10 g/ml streptomycin at 37°C in 5% CO2 in a water-saturated atmosphere.

### Reagents and antibodies

Epigallocatechin gallate (EGCG) and CCK8 reagent were perchased from Sigma-Aldrich (St. Louis, MO, USA). Primers for MDR-1, P-gp, GAPDH were synthesized from company(Thermo Fisher Scientific, Shanghai). TRI reagent was from Solgent (Life, Shanghai). AmpliTaq DNA polymerase was obtained from Roche Inc (Indianapolis, IN). Enzyme-linked immunosorbent assay (ELISA) kit for VEGF was obtained from R&D Systems (Minneapolis, MN, USA). For immunoblotting, specific antibodies against MDR-1, P-gp, GST-π, TFAP2A, p-TFAP2A, VEGF, GAPDH and the secondary antibodies were obtained from Santa Cruz Biotechnology Inc.

### Establishment of 5-fluorouracil resistant cells

The variants of SGC-7901 and MGC-803 cells, SGC-7901/FU and MGC-803/FU, which are 5-Fluorouracil resistant of each cells, were established by step-wise exposure of the parental cells to escalating concentrations of 5-Fluorouracil, ranging from 1uM to 10mM for more than 6 months.

### RT-PCR

Cells (2×10^5^) were seeded in 100 mm culture dish and grown overnight at 37°C and then treated with the indicated concentrations of EGCG for the 48 hours. Total RNA was extracted using TRI reagent and subjected to the cDNA synthesis and PCR. The specific primers were as follows: MDR-1, sense 5’-AACCTTCAACTCCTGCCTTCTCG-3’ and antisense 5’-CAGCTTCTCCTTCAGCTCTTCAC-3’; P-gp, sense 5’-GTGTGTGGCTGACTTCGGAC-3’ and antisense 5’-CACGTCCTCCATACACTCCG-3’; GAPDH, sense 5’-GGAGCCAAAAGGGTCATCAT-3’ and antisense 5’-GTGATGGCATGGACTGTGGT-3’.

### Western blot analysis

Cells were treated with the indicated concentration of EGCG for 24 hours. Total cell lysates were prepared from those cells using lysis buffer (1% Triton X-100, 50 mM Tris (pH 8.0), 150 mM NaCl, 1 mM PMSF, 1 mM Na3VO4, and protease inhibitor cocktail). Protein concentrations were determined using Bio-Rad protein assays (Sigma-Aldrich, Shanghai, China)). Proteins from cell lysates (40-80 μg) were separated on 10% SDS-PAGE, and electrotransferred to PVDF membranes. Membranes were blocked for 60 minutes at room temperature in Tris buffer saline 0.1% Tween-20 (TTBS) containing 7% non-fat dry milk, and then incubated with TTBS containing a primary antibody for 4 hr at room temperature. After 3× 5 min washes in TTBS, membranes were incubated with peroxidase-conjugated secondary antibody for 1 hr. Following 3 additional 10 min washes with TTBS, protein bands of interest were visualized using an enhanced chemiluminescence detection system.

### Colony formation assay

Cells were seeded in 35 mm culture dishes (400 cells/dish) and allowed to grow for 10 to 14 days in the presence of or absence of EGCG to form colonies. Colonies of more than 50 cells were visualized by crystal violet staining and images were taken by RAS 4000 Image Analysis System.

### Cell viability assay

The viability of cells was measured using Cell Counting Kit-8 assay kit (Sigma-Aldrich, Shanghai, China). Cells (1×10^3^ cells/well) were seeded in 96 well plates and grown overnight at 37°C and then treated with the indicated concentrations of EGCG or 5-fluorouracil for the indicated hours. At the end of treatment, 10 μl of CCK-8 solution was added and then incubated for 4 hours. The absorbance at 450 nm was measured using a microplate reader (Model 680 microplate reader, Bio-Rad Laboratories). Values are the mean±SD for triplicate wells and normalized to that of control group to determine the % of viability.

### ELISA analysis for VEGF

To determine VEGF level, cells were incubated for 48 hours in the presence or absence of EGCG. After treatment, conditioned media were harvested and assayed for VEGF by ELISA according to the manufactures’ protocol [30]. The data are the representative of at least three independent experiments.

### Xenograft mouse model

Female BALB/c nude mice, 4–6 weeks of age, were purchased from Shanghai Animal Laboratory Center (Shanghai, China) and maintained in appropriate sterile filter capped cages in the Experimental Animal Center at Guangzhou Medical University. Exponentially growing SCG7901/FU cells (5 × 106) were injected subcutaneously into the dorsum of the mice. After tumor transplantation for one week, the body weight and the tumor size were recorded twice a week. The length and width of tumor were measured using a caliper, and the volumes were calculated by the following formula: volume (mm3) = length × width^2^/2. On the 8th day after transplantation, 20 mice were randomized into 4 groups (5 mice of each group) and treated as follows: Control (normal saline, 0.1ml/10kg), EGCG (25 mg/kg), 5-FU (20 mg/kg), EGCG (25 mg/kg) and 5-FU (20 mg/kg). EGCG was administered twice a week and 5-FU was given twice a week by intraperitoneal injection. After treatment for 4 weeks, xenograft tumors were isolated from mice. A portion of the tumors tissue was fixed in 4% paraformaldehyde for histological study. No mice were sacrificed before the end of the experiment and the mice were euthanized by cervical dislocation.

### Immunohistochemistry (IHC)

Immunohistochemistry was performed to study VEGF, MDR1, P-gp and p-TFAP2A proteins expression in Xenograft. In brief, paraffin-embedded specimens were cut into 4 μm sections and baked at 65°C for 30 min. The sections were de-paraffinized with xylenes, rehydrated, submerged into citrate buffer and microwaved for antigenic retrieval. They were then treated with 3% hydrogen peroxide in methanol to quench the endogenous peroxidase activity, followed by incubation with 3% BSA to block the nonspecific binding. Rabbit polyclonal antibody was incubated with the sections overnight at 4°C. For negative controls, the primary antibody was replaced by rabbit serum. After washing, the tissue sections were treated with biotinylated anti-rabbit secondary antibody (Cell Signaling Technology, USA), and further incubated with streptavidin horseradish peroxidase complex (Cell Signaling Technology, USA). The degree of immunostaining of formalin-fixed and paraffin-embedded sections was reviewed and scored by two independent observers. The proportion of the stained cells and the extent of the staining were used as criteria of evaluation.

### Statistical analysis

All analysis were performed using Graph Pad Prism Ver. 6.0 (GraphPad Software Inc. San Diego, CA). Data are presented as the mean±SD of triplicate samples or at least three independent experiments. All data with statistical significance were indicated when P <0.05. Statistical comparisons between control and treatment groups were determined using unpaired Student’s t-test or one-way ANOVA.
